# From transients to permanent residents: the existence of obligate aerobic microorganisms in the goat rumen

**DOI:** 10.3389/fmicb.2024.1325505

**Published:** 2024-01-22

**Authors:** Rongjiao Wang, Dan Huang, Changguo Chen, Dingzhou Song, Hongen Peng, Min He, Xiujun Huang, Zhonghua Huang, Bin Wang, Hai Lan, Ping Tang

**Affiliations:** Panzhihua Academy of Agricultural and Forestry Sciences, Panzhihua, China

**Keywords:** rumen, anaerobic microorganisms, aerobic microorganisms, volatile fatty acid rumen, volatile fatty acid

## Abstract

The rumen serves as a complex ecosystem, harboring diverse microbial communities that play crucial ecological roles. Because previous studies have predominantly focused on anaerobic microorganisms, limited attention has been given to aerobic microorganisms in the goat rumen. This study aims to explore the diversity of aerobic microorganisms in the rumen and understand their niche and ecological roles. Rumen fluid samples were collected from 6 goats at different time points post-morning feeding. pH, NH_3_-N, and volatile fatty acid (TVFA) concentrations were measured, while *In vitro* cultivation of aerobic microorganisms was performed using PDA medium. Internal Transcribed Spacer (ITS) and 16S sequencing unveiled microbial diversity within the rumen fluid samples. Evidence of obligate aerobic microorganisms in the goat rumen suggests their potential contribution to ecological functionalities. Significantly, certain aerobic microorganisms exhibited correlations with TVFA levels, implying their involvement in TVFA metabolism. This study provides evidence of the existence and potential ecological roles of obligate aerobic microorganisms in the goat rumen. The findings underscore the significance of comprehensively deciphering goat rumen microbial communities and their interactions, with aerobes regarded as permanent residents rather than transients. These insights form a solid foundation for advancing our understanding of the intricate interplay between goat and their aerobic microorganisms in the rumen.

## 1 Introduction

The rumen is a complex microbial ecosystem that harbors a diverse array of microorganisms, establishing an intricate mutualistic system with animals (Wallace et al., 2019; Wilkinson et al., 2020; Peng et al., 2021; Ramos et al., 2022). The microbial population within the rumen plays a crucial role in converting plant material of relatively low nutritional value into vital compounds readily absorbed by the host animal (Shabat et al., 2016; Wallace et al., 2019; Aggarwala et al., 2021). Both physiologists and nutritionists recognize the pivotal role of rumen microorganisms in digesting fibrous feed and providing essential nutrients to the host animal (Church, 1993; Lam et al., 2018; Li et al., 2019; Wallace et al., 2019). Studies have also demonstrated that the rumen microbiota in ruminants may be affected by multiple factors such as host variety or species (Guan et al., 2008; Henderson et al., 2015; Li et al., 2019).

While the rumen is primarily characterized as an anaerobic environment (Wilkinson et al., 2020), it is not strictly obligate as trace amounts of oxygen enter during feeding and regurgitation. Consequently, aerobic microorganisms have the potential to survive within this ecosystem. It is precisely because of the rumen’s apparent “anaerobic nature,” that previous research has predominantly focused on anaerobic microorganisms. However, there have been studies detecting the presence of aerobic microorganisms in the rumen of different ruminant species (Fonty et al., 1987; MINATO et al., 1992). Early studies identified the presence of spores and hyphae of obligate aerobic fungi in the rumen through morphological examination. The specific role of these obligate aerobic fungi was unclear due to the limited research methods available at that time. At the same time, researchers also reported the rapid colonization of aerobic and facultative anaerobic microbial groups in the rumen of newborn ruminants (Jami et al., 2013).

Furthermore, sequencing studies have identified the presence of some aerobic microorganisms in the rumen. In 2012, Patrícia et al. discovered a significant presence of Aspergillus in the rumen (Almeida et al., 2012). In 2020, Elnaz et al. detected aerobic fungi in the rumen using ITS sequencing (Azad et al., 2020). In the same year, Xing et al. detected Fusarium and Aspergillus in rumen fluid (Xing et al., 2020). R.F. Neto’s study in 2020 indicated the presence of Trichoderma in the rumen and emphasized its ability to survive (Fabino Neto et al., 2020). In 2022, our research group detected aerobic fungi in the rumen of buffaloes (Wang et al., 2022). Researchers speculate that these fungi may represent opportunistic populations residing in feed and other substrates and may not directly participate in rumen fermentation or their specific role remains unknown.

This study was premised on the existence of aerobic microorganisms in the goat rumen, which are capable of surviving until the subsequent feeding and rumination of ruminants. Considering this premise, we investigated the microbial diversity of the goat rumen at various time points. If aerobic microorganisms within the rumen undergo multiplication and growth in this relatively anaerobic environment, it may necessitate a reevaluation of the composition of rumen microorganisms. This observation challenges the notion that aerobic microorganisms in the goat rumen are merely “transient microorganisms” and emphasizes their role as “resident microorganisms.”

## 2 Materials and methods

### 2.1 Experimental animals and experimental design

Test animals were six female Nubian black goats that had never been pregnant. (Average body weight: 30 ± 0.5 kg. 6 months old). Rumen fluid samples were collected on day 30 during the sampling period between 2, 4, 8, and 0 (14) hours after the morning feeding was delivered to goats. Rumen fluid was extracted via the mouth using a stomach tube with a rumen vacuum sampler at the corresponding point in time.

Roughage was king grass and was supplemented with cornmeal 0.1 kg per goat per day. The experimental animals were fed twice a day at 8:00 a.m., and 6 p.m.

### 2.2 Chemical analysis

Samples of roughage grass were ground in a mill to pass a 1-mm screen and analyzed for ether dry matter (DM), neutral detergent fiber (NDF), acid detergent fiber (ADF), crude ash (Ash), crude protein (CP), ether extract (EE), calcium (Ca), phosphorus (P) (Licitra et al., 1996; al-Suwaiegh et al., 2002).

Rumen fluid PH was measured in time by PHS-3C pH meter after rumen fluid collection. The acidity meter was preheated and calibrated half an hour before the formal determination. The phenol-hypochlorous acid colorimetric method was used to measure NH_3_-N. Rumen fluid after centrifugation at 12000 g for 20 min, 40 mL of the supernatant was added to 2.5 mL of phenol chromogenic agent and 2.0 mL of sodium hypochlorite reagent, respectively. After that, the supernatant was thoroughly mixed by shaking and placed in a 37°C water bath for 30 min. With a visible spectrophotometer at 550 nm, the supernatant was analyzed colorimetrically. Standard curves were drawn by NH_4_Cl standard solution (y = 0.1613x + 0.1039 R^2^ = 0.9063). NH_3_-N concentration was calculated based on colorimetric results and standard curves. The extraction of volatile fatty acids involved diluting the rumen fluid sample with ddH_2_O, adding 15% phosphoric acid, isohexanoic acid, and diethyl ether homogenate before centrifugation. Chromatographic determinations were carried out using an Agilent HP- INNOWAX Capillary column (30 m*0.25 mm ID*0.25 mm) (Wang et al., 2023).

### 2.3 Microbial culture

Rumen fluid collected before feeding was used for inoculation culture, specifically 0 h post-feeding (14 h after the previous feeding). The whole microbial culture was done by PDA medium without the addition of drugs. After inoculation under aseptic conditions, the petri dishes were placed in a conventional incubator at 25 degrees Celsius for 7 days. Microorganisms coenobium were observed microscopically after coating the plates.

### 2.4 Amplification and sequencing

DNA extraction: Cell lysis of rumen fluid was achieved by beading in the presence of 4% (w/v) sodium dodecyl sulfate (SDS), 500 mM NaCl, and 50 m EDTA. The buffer acts to protect the released DNA from degradation by DNase, which is very active in rumen fluid samples. After beading, impurities and SDS were removed by ammonium acetate precipitation, and nucleic acid was removed by isopropanol precipitation. Zymo Research BIOMICS DNA Microprep Kit was used for sample gDNA purification (Wanapat and Cherdthong, 2009; Kuczynski et al., 2011).

Additive sequencing with Illumina sequencing technology was used in the experiment. The 16S rRNA of prokaryotes, the ITS gene of fungi, or specific functional genes can be used in taxonomic identification (Ekelund et al., 2006). The following universal primers were applied for the amplification of the V4 region of the 16S rRNA gene. Primer5’-3′: 515F (5’-GTGYCAGCMGCCGCGGTAA-3′) and 806R(5‘-GGACTACHVGGGTWTCTAAT-3′). The conditions of the real-time PCR assays were as follows: for 16S rRNA: 1 min at 94°C for initial denaturation (1 cycle), 20 s at 94°C for denaturation, 30 s at 54°C for annealing, and 30 s at 72°C for extension (30 cycles), and a final extension period of 10 min at 72°C. Three separate PCRs for each sample were pooled for processing.

The following universal primers were applied for the amplification of the ITS3 region of the ITS gene. Primer5`-3`: ITS3 (5`-GATGAAGAACGYAGYRAA-3`) and ITS4(5`-TCCTCCGCTTATTGATATGC-3`). The PCR protocol comprised an initial denaturation at 94°C for 1 min, followed by a denaturation step at 94°C for 20 s, annealing at 50°C for 30 s, and extension at 72°C for 30s for 25–30 cycles. The protocol concluded with a final extension step at 72°C for 5 min, followed by holding at 4°C. Three independent PCR replicates were performed for each sample, and the PCR products in the linear phase of amplification were pooled in equal amounts and used for subsequent library construction.

Libraries were constructed using NEBNext Ultra II DNA Library Prep Kit for Illumina from NEW ENGLAND BioLabs. PE250 was used for high-throughput sequencing, and the sequencing Kit was Illumina Hiseq Rapid SBS Kit v2(FC-402-4023 500 Cycle).

### 2.5 Data analysis

The original offline data obtained by sequencing were spliced and filtered to obtain high-quality target sequences for subsequent analysis. Subsequent operation of bioinformatics research (http://www.drive5.com/usearch) and QIIME completed, such as statistics and drawing mainly using R, Python, and Java (Brown et al., 2015).

Data quality control: Splice a two-ended sequence using FLASH. Each sample sequence was isolated from raw reads based on the barcode, and the barcode sequence was truncated. Then use QIIME for quality control. Filter out sequences with an average mass of less than 25. The sequence length of less than 200 bp was removed. The sequence with more than 2 fuzzy bases (N) was removed. The Uchime algorithm and gold database were used to remove chimeras, and effective data Effective Tag was obtained. Community composition analysis: R language was used for various data conversions and ggplot2 package mapping.

Difference species analysis: LefSe analysis using LefSe tools (https://bitbucket.org/biobakery/biobakery/wiki/Home). randomForest package using R language is used for random forest analysis. Metastas analysis using R language script, calculation steps (https://journals.plos.org/ploscompbiol/article?id=10.1371/journal.pcbi.1000352) for reference.

Community function prediction: Cluster and annotate the sequencing data of the 16S rRNA and ITS gene based on the SILVA database, and linearly transform the calculated results based on the pre-calculated correlation matrix to obtain the microbial classification spectrum based on the KEGG database. The results were corrected according to the copy number of the 16S rRNA and ITS gene in the genome of different bacteria/ fungi in NCBI. The classification information was linearly predicted based on the functional gene profiles of microorganisms in the KEGG database.

Excel was used to organize the test data, and SPSS 22.0 and Origin 2021 were used to conduct variance analysis, unary linear regression analysis, correlation analysis between variables, curve regression analysis, and Duncan’s multiple comparison test. Some software and database version information: QIIME v1.9.0, Usearch 10.0.240, R language: 3.6.0, Python: 3.7.4, SILVA database: 132. The results were expressed as mean ± standard deviation. *p* < 0.05 was used as the difference significance criterion, and *p* < 0.01 was used as the criterion of extremely significant difference. Records will be accessible with the following link after the indicated release date: http://www.ncbi.nlm.nih.gov/bioproject/996308. BioProject ID: PRJNA996308. http://www.ncbi.nlm.nih.gov/bioproject/997758. BioProject ID: PRJNA997758.

## 3 Results

### 3.1 The internal environment of the goat rumen

**Figure 1:**
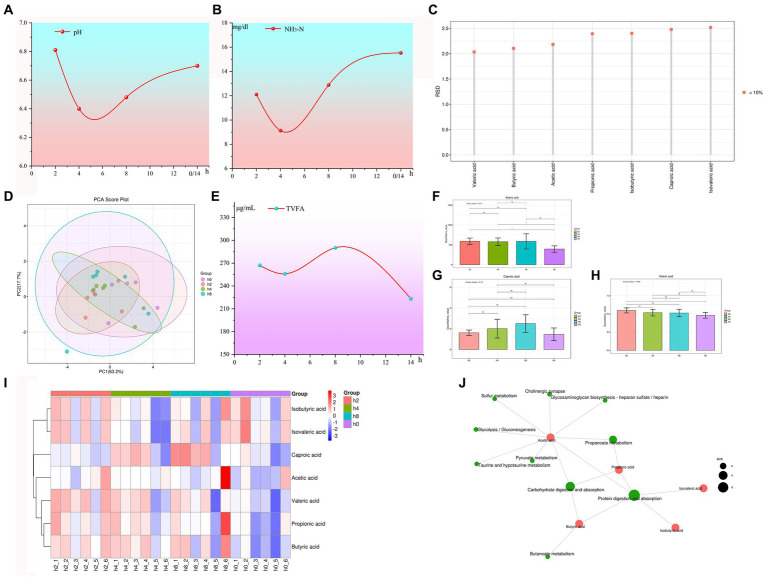
The internal environment of the goat rumen changes with time after feeding. Note: **(A)** Rumen fluid pH changes with time after feeding. **(B)** NH_3_-N concentration changes with time after feeding. **(C)** The reproducibility of quality control (QC) samples. **(D)** Principal component analysis of volatile fatty acids. **(E)** Total volatile fatty acid (TVFA) concentration with time after feeding. **(F)** Concentration levels of butyric acid with time after feeding. **(G)** Concentration levels of valeric acid with time after feeding. **(H)** Concentration levels of caproic acid with time after feeding. **(I)** Correlation heat maps of volatile fatty acids with different time points. **(J)** KEGG metabolite molecular network diagram.

Based on the data presented in Figures 1A,B, observations of goat rumen fluid pH and NH_3_-N concentration levels revealed a decreasing-then-increasing trend following cessation of feeding. This pattern was consistently observed for both characteristics, with pH recording higher levels at 2 and 14 h after morning feeding, as well as lower levels at 4 and 8 h.

Similarly, NH_3_-N concentration registered a significantly elevated level at the 8 and, 14 h marks following feeding while remaining decreased after 2 and 4 h. This consistent relationship mirrors the impact that NH_3_-N has on altering the acidity levels of rumen fluid. Additionally, Figure 1B also illustrated a correlation between rumen fluid acidity and NH_3_-N concentration to some degree.

The reproducibility of quality control (QC) samples is often assessed using the relative standard deviation (RSD), with a requirement for RSD values to be below 15%. Figure 1C presents data that consistently yields RSD values below 2.6%, which is significantly lower than the required threshold. These findings demonstrate a high level of reproducibility and accuracy in the experimental data obtained.

As shown in Figure 1D, the results of the principal component analysis of volatile fatty acids showed that the first principal component accounts for 63.2% of the variance, with the second principal component explaining an additional 17.7% of the total variability.

As shown in Figure 1E, the concentration of total volatile fatty acids (TVFA) reached its peak at 8 h after morning feeding and was lowest at 14 h. As shown in Figures 1F–H. The concentrations of butyric and caproic acid initially increased and then decreased with the duration of morning feeding time, reaching their highest point at 8 h. Figure 1I, Heatmap analysis revealed a decrease in the concentrations of propionic, valeric, acetic, and caproic acid with increasing duration of morning feeding time. As shown in Figure 1J, acetic acid plays a primary role in carbohydrate digestion, absorption, propanoate metabolism, and associated metabolic pathways.

### 3.2 *In vitro* culture of goat rumen microorganisms

**Figure 2:**
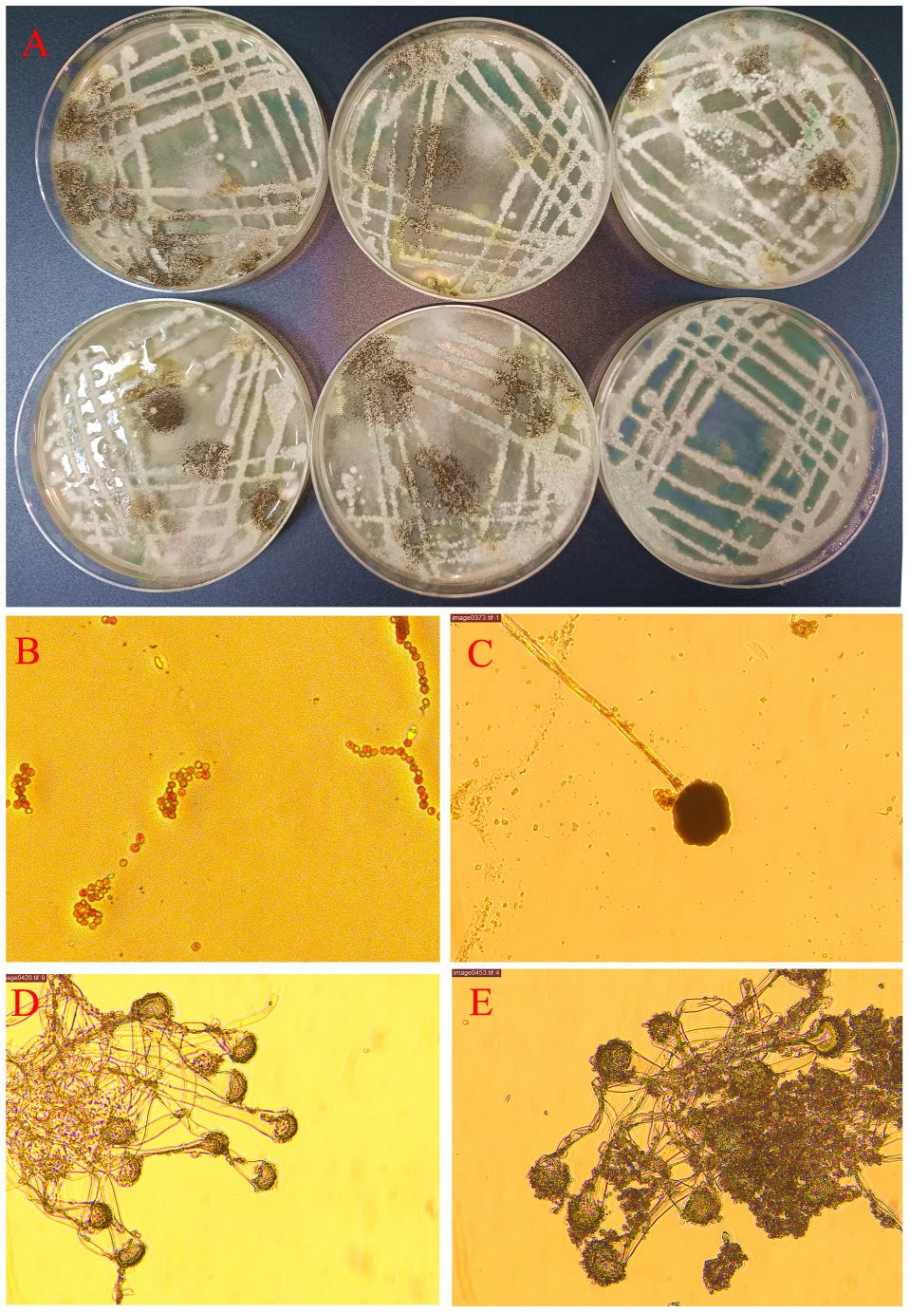
Goat rumen microorganisms were cultured *in vitro* under normal air conditions. Note: **(A)** Images of Petri dishes were obtained 7 days after inoculation of rumen fluid for aerobic culture. **(B)** Fungal structures suspected to be Candida were under a microscope at 400 magnification. **(C)** Fungal structures suspected to be Mucorales were under a microscope at 400 magnification. **(D,E)** Fungal structures suspected to be Aspergillus under a microscope at 400 magnification.

Rumen fluid collected before feeding was used for inoculation culture, specifically 0 h post-feeding (14 h after the previous feeding), using solid media for culture under aerobic conditions. The experimental findings are visually presented in Figure 2A, clearly demonstrating a substantial proliferation of colonies on the media following a 7-day incubation period.

Microscopic observations revealed the presence of a considerable number of spores and hyphae (Figure 2B). The most frequently observed fungal structures under microscopic examination resembled Candida, suggesting the presence of yeast-like fungi (Figure 2B). Additionally, examination revealed fungal structures resembling mold-like mucor (Figure 2C). Furthermore, examination under the microscope identified fungal structures resembling Aspergillus species (Figures 2D,E). It is worth noting that the predominance of these three fungal structures could be attributed to limitations imposed by *in vitro* culture conditions. Of particular interest is the fact that the genus Aspergillus is obligate aerobes.

### 3.3 Bacterial diversity in the goat rumen

**Figure 3:**
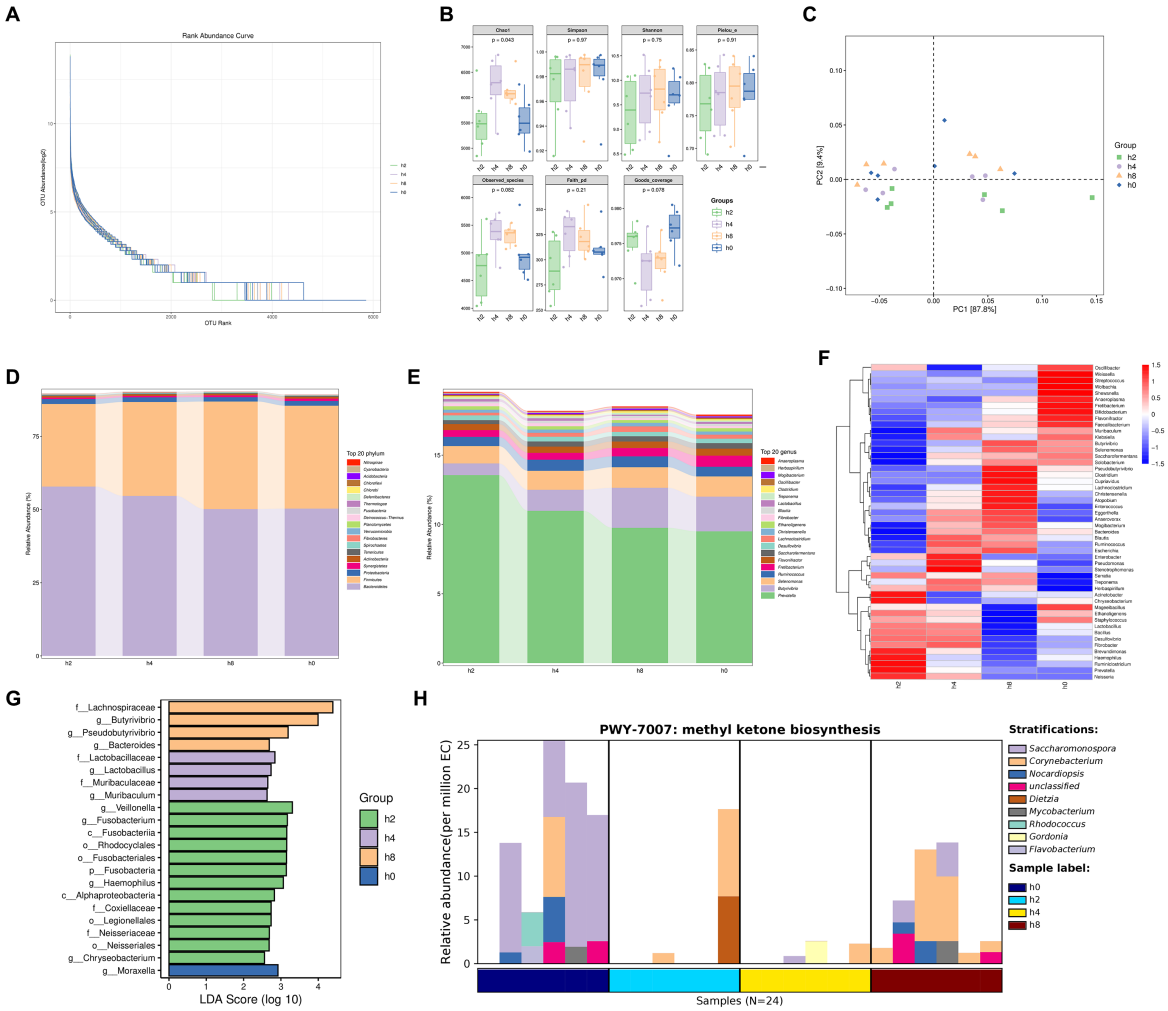
Bacterial diversity in the goat rumen fluid at different points in time. Note: **(A)** Rank abundance curve, **(B)** The α diversity of bacteria, **(C)** Principal component analysis. **(D)** Bacterial composition of relative abundance of phylum level in rumen fluid at different points in time. **(E)** Bacterial composition of relative abundance of genera level in rumen fluid at different points in time. **(F)** Correlation of bacterial composition with time after ingestion. **(G)** Bacterial communities with significant differences at different points in time. **(H)** Methyl ketone biosynthesis shows that the main bacteria groups.

Based on the results depicted in Figure 3A, it was observed that the increase in the number of samples did not lead to a subsequent increase in OUT abundance. Figure 3B displayed higher chao1 values for the h4 group compared to the other groups, with h8, h0, and h2 following in ascending order. The principal component analysis conducted in Figure 3C highlighted that PC1 explained 87.8% of the variances observed.

As observed in Figures 3D,E, the relative abundance of Bacteroidetes gradually decreased over time following a meal. In contrast, the relative abundance of Firmicutes initially increased and then declined, reaching its peak 8 h after morning feeding. The relative abundance of Prevotella exhibited a gradual decrease with increasing time after morning feeding. Similarly, the relative abundances of Butyrivibrio, Selenomonas, and Lachnoclostridium showed a rising-then-declining trend over time, reaching their highest levels 8 h after morning feeding. Conversely, the relative abundance of Fretibacterium increased gradually with time following morning feeding.

As depicted in Figure 3F, the relative abundance of rumen bacteria at both phylum and genus levels exhibited regular changes with increasing time after morning feeding.

Figure 3G highlights significant differences in bacterial communities at four-time points. These differences include f_Lachnospiraceae, g_Butyrivibrio, g_Pseudobutyrivibrio, g_Bacteroides, f_Lactobacillaceae, g_Lactobacillus, f_Muribaculaceae, g_Muribaculum, g_Veillonella, g_Fusobacterium, c_Fusobacteria, o_Rhodocyclales, o_Fusobacteriales, p_Fusobacteria, g_Haemophilus, c_Alphaproteobacteria, f_Coxiellaceae, o_Legionellales, f_Neisseriaceae, o_Neisseriales, and g_Chryseobacterium, g_Moraxella.

In Figure 3H, the synthesis of methyl ketones reveals that Corynebacterium and Dietzia were the main bacterial groups observed 2 h after a meal. At 4 h after eating, the main groups were Corynebacterium, Gordonia, and Flavobacterium. After 8 h of morning feeding, the main bacterial groups were Corynebacterium, Saccharomonospora, Nocardiopsis, and other unclassified species. Flavobacterium, Corynebacterium, Nocardiopsis, Rhodococcus, and other unclassified species were the main bacterial groups observed immediately (0 h) after morning feeding.

Overall, the relative abundance of rumen bacteria consistently exhibited patterns of increase or decrease over time following feeding. While the majority of the identified bacteria were anaerobic, some aerobic bacteria, such as Flavobacterium, Nocardiopsis, and Moraxella, were also detected within the rumen microbiome.

### 3.4 Fungi diversity in the goat rumen

**Figure 4:**
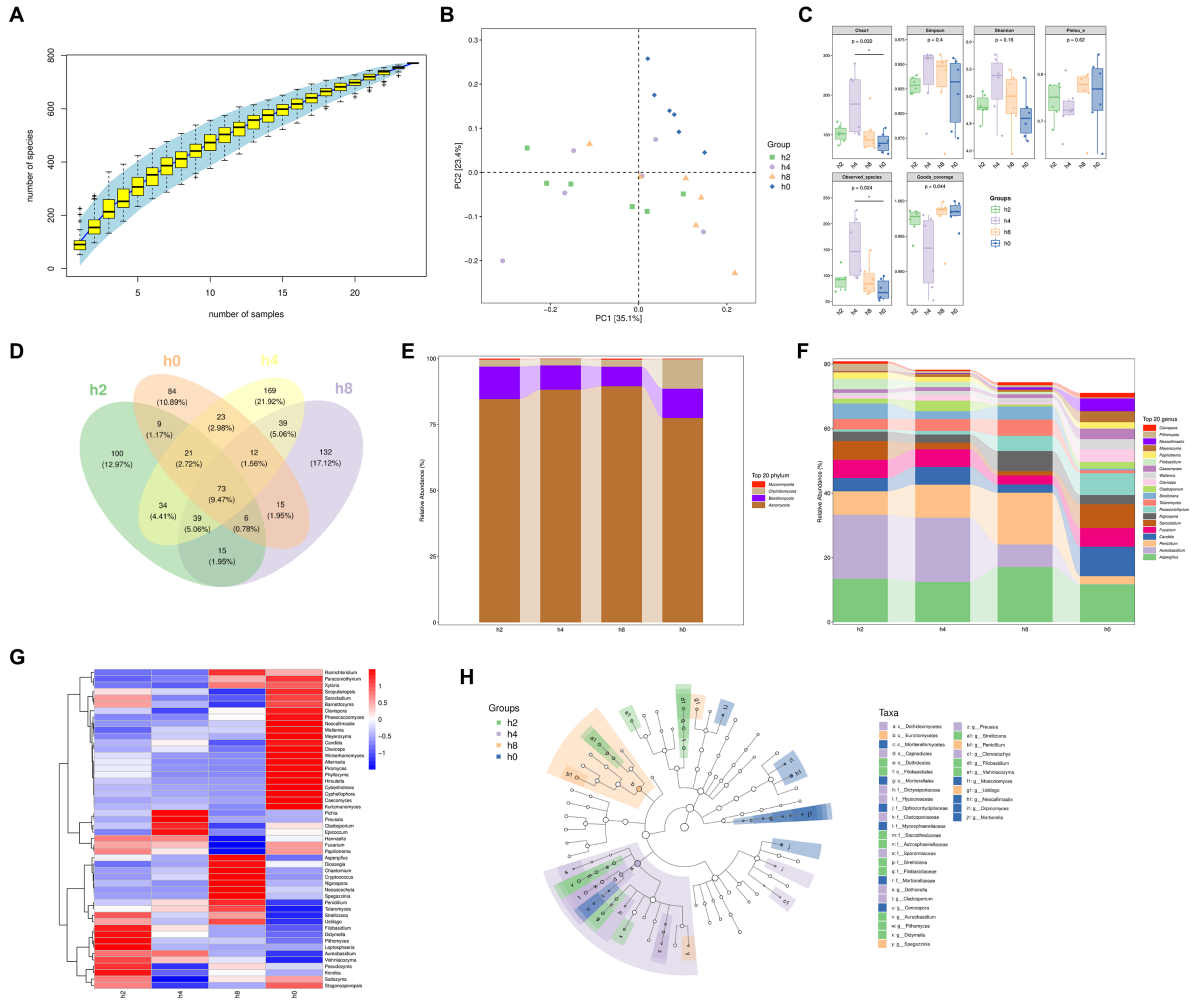
Fungi composition and differences in the goat rumen fluid. Note: **(A)** Rank abundance curve, **(B)** Principal component analysis, **(C)** The α diversity of bacteria, **(D)** Venn diagram showing numbers of taxonomic assignments, **(E)** Fungi composition of relative abundance of phylum level in rumen fluid at different points in time, **(F)** Fungi composition of relative abundance of genera level in rumen fluid at different points in time, **(G)** Correlation of Bacterial composition with time after ingestion, **(H)** The branching diagram of differential species.

Based on the results presented in Figures 4A,B, as the number of samples increased, the number of species identified also steadily rose. However, the rate of increase began to level off once the sample size reached 24. Additionally, the principal component analysis revealed that PC1 accounted for 35% of the variance, with the h0 group appearing clustered in the upper right quadrant.

Figure 4C shows that both Chao1 and observed species counts were significantly higher in group h4 compared to other groups. Moreover, the Simpson and Shannon indices showed the highest values in group h4, followed by group h8, indicating a greater diversity of microbial species in h4 than in the other groups.

Figure 4D illustrates that the Venn diagram depicted specific OUT groups, whereby group h2 had 100 species unique to it, while groups h0, h4, and h8 had 84, 169, and 132 unique species, respectively.

Moreover, the fungal composition within the goat rumen environment, as shown in Figure 4E, primarily belonged to the Ascomycota, Basidiomycota, Chytridiomycota, and Mucoromycota phyla. Interestingly, their relative abundance patterns exhibited variations after the morning feeding time. The relative abundance of Ascomycota initially increased before decreasing, whereas the relative abundances of Basidiomycota and Chytridiomycota decreased before ultimately increasing as the morning feeding time lengthened.

**Figure 5:**
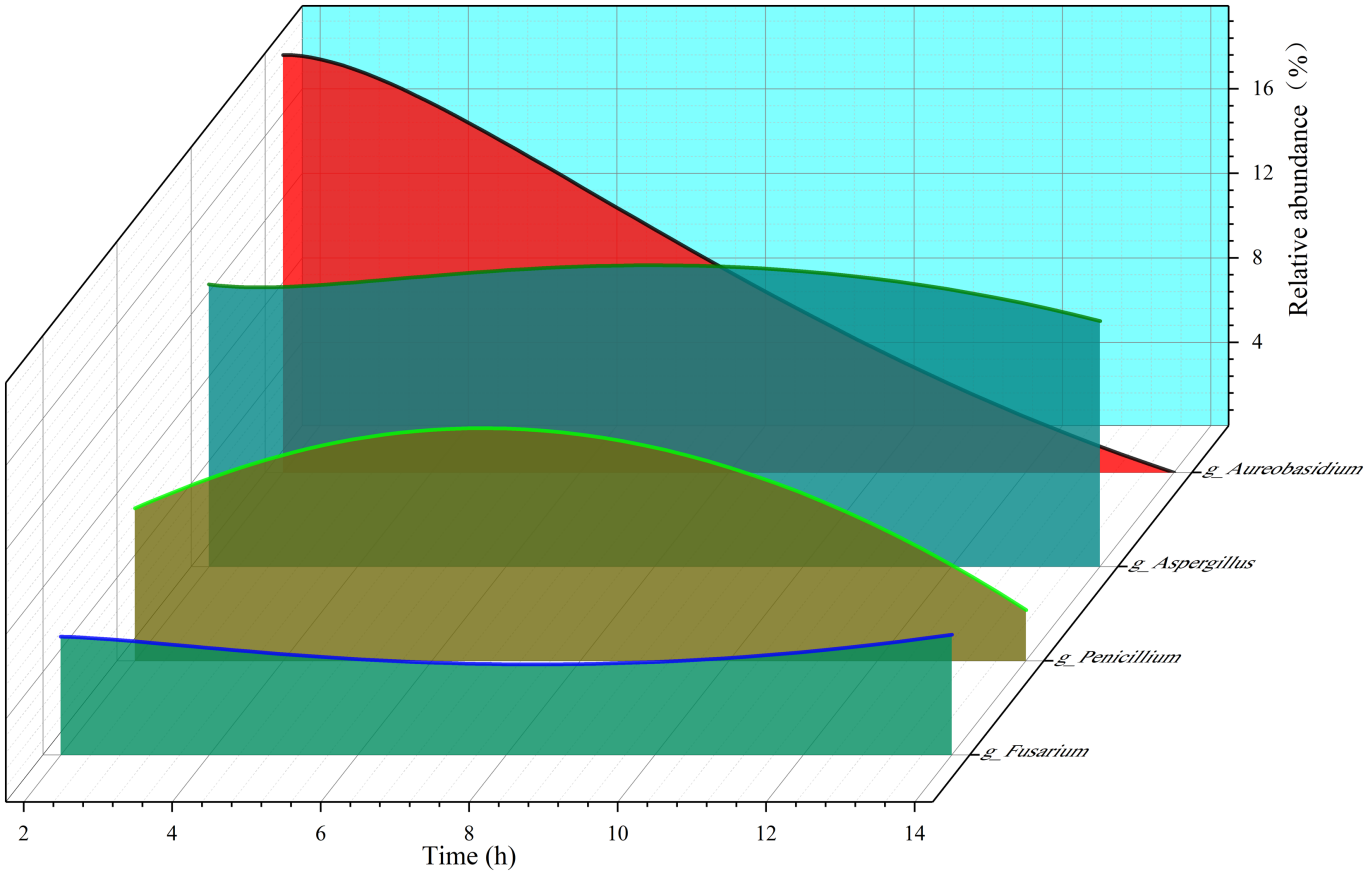
The relative abundance of the four fungi changed with the extension of time after morning feeding.

As shown in Figure 4F, the goat rumen fungi mainly consist of Aspergillus, Aureobasidium, Penicillium, Candida, Fusarium, arocladium, Nigrospora, Paraconiothyrium, Talaromyces, Strelitziana, Cladosporium, Claviceps, Wallemia, Caecomyces, Filobasidium, Papiliotrema, Meyerozyma, Neocallimastix, Pithomyces, Clavispora in genus level. By examining Figures 5E–G together, it becomes evident that the time after morning feeding influences the relative abundance of various bacterial communities in different patterns.

The branching diagram of differential species, as presented in Figure 4H, highlights that several fungal taxa showed significant differences at different time points. These included c_Dothideomycetes, c_Eurotiomycetes, c_Mortierellomycetes, o_Capnodiales, o_Dothideales, o_Filobasidiales, o_Morierellales, f_Dictyosporiaceae, f_Hypocreaceae, f_Ophiocordycipitaceae, t_Cladosporiaceae, f_Mycospharellaceae, f_Saccotheciaceae, f_Astrosphaerillaceae, f_Sporormiaceae, f_Strelitziana, f_Filobasidiaceae, f_Mortierellaceae, g_Dothiorella, g_Cladosporium, g_Cercospora, g_Aureobasidium, g_Pithomyces, g_Didymella, g_Spegazzinia, g_Preussia, g_Strelitziana, g_Penicillium, g_Clonostachys, g_Filobasidium, g_Vishniacozyma, g_Moesziomyces, g_Ustilago, g_Neocallimastix, g_Orpinomyces, and g_Mortierella.

### 3.5 Correlation between volatile fatty acids and goat rumen microorganisms

**Figure 6:**
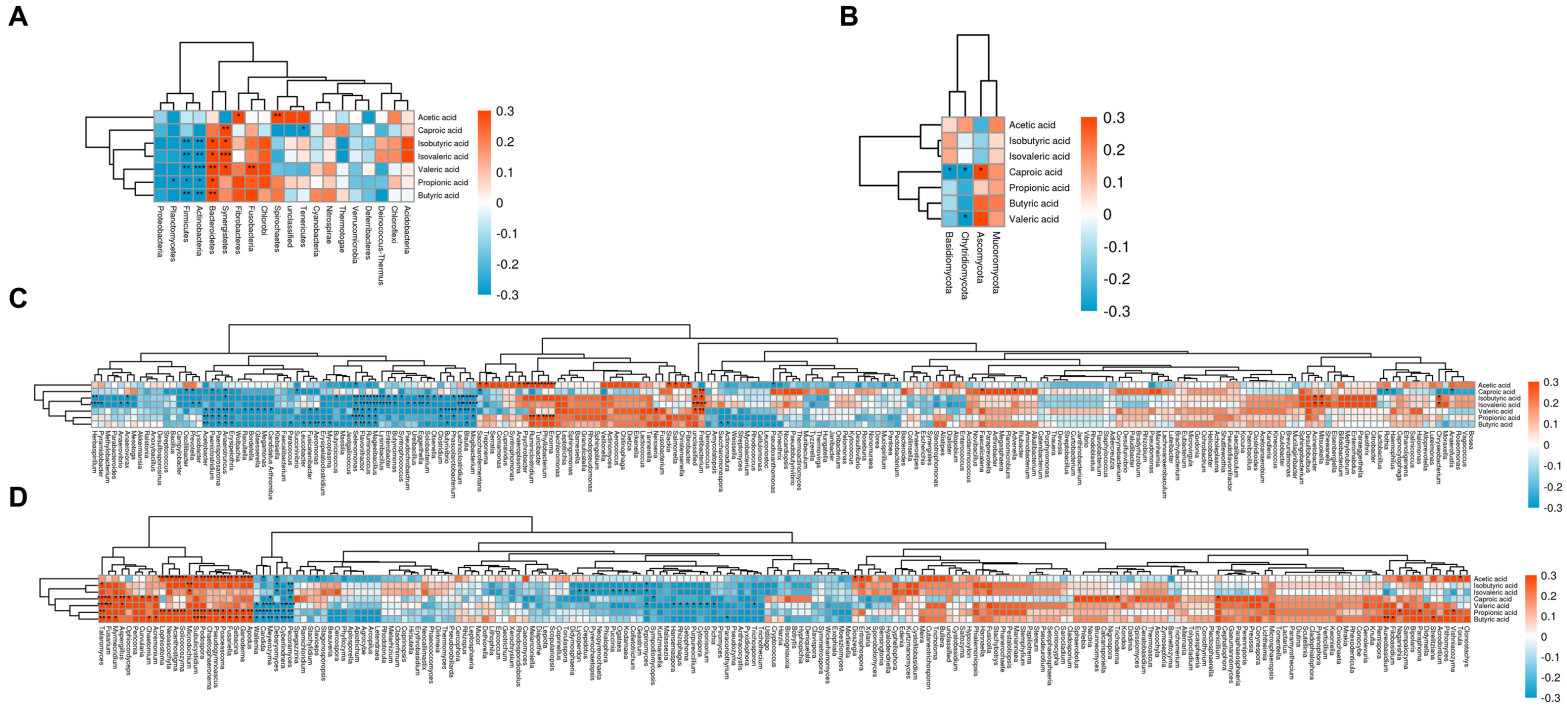
Correlation between volatile fatty acids and goat rumen microorganisms. Note: **(A)** The phylum level of bacteria was correlated with VFA. **(B)** The phylum level of fungi was correlated with VFA. **(C)** The genus level of bacteria was correlated with VFA. **(D)** The genus level of fungi was correlated with VFA.

Figure 6A reflected that the bacterial phyla significantly associated with volatile fatty acids in the goat rumen included Fusobacteria, Fibrobacteres, Synergistetes, Bacteroidetes, Actinobacteria, Firmicutes, Planctomycetes, Spirochaetes, and Tenericutes. Similarly, Figure 6B demonstrated that Ascomycota, Chytridiomycota, and Basidiomycota were the fungal phyla significantly correlated with volatile fatty acids in the goat rumen.

Figure 6C illustrated that 63 bacterial genera significantly correlated to volatile fatty acids in the goat rumen included Pyramidobacter, Herbaspirillum, Anaerovorax, Anaerovibrio, Erysipelothrix, Anaerotruncus, Paenisporosarcina, Paeniclostridium, Acetobacter, Lysobacter, Prevotella, Oscillibacter, Butyricicoccus, Mycoplasma, Erysipelatoclostridium, Aeromonas, Fusicatenibacter, Leucobacter, Succinivibrio, Paracoccus, Treponema, Saccharofermentans, Mogibacterium, Blautia, Lachnoclostridium, Phascolarctobacterium, Butyrivibrio, Clostridium, Olsenella, Solobacterium, Eggerthella, Ureibacillus, Pseudochrobactrum, Syntrophococcus, Butyricimonas, Enterobacter, Lysinibacillus, Mageeibacillus, Ruminococcus, Flavonifractor, Selenomonas, Enorma, Phyllobacterium, Turicibacter, Ruminiclostridium, Psychrobacter, Anaeroplasma, Neisseria, Fretibacterium, Unclassified, Fibrobacter, Salmonella, Slackia, Actinomadura, Saccharomonospora, Pseudoxanthomonas, Faecalicatena, Advenella, Mitsuokella, Acinetobacter, Rothia, Anaerofustis, and Chryseobacterium. It is noteworthy that some of these bacteria genera are strictly aerobic or facultatively anaerobic.

Figure 6D revealed that there were 59 fungal genera that significantly to volatile fatty acids in the goat rumen including Pecoramyces, Cyberlindnera, Debaryomyces, Meyerozyma, Candida, Wallemia, Apodus, Ganoderma, Sebacina, Fusariella, Poaceascoma, Pseudogymnoascus, Chaetosphaeronema, Podospora, Subulicystidium, Microdochium, Solicoccozyma, Acanthostigma, Neoascochyta, Lophiostoma, Acremonium, Chaetomium, Curvularia, Ophiocordyceps, Aspergillus, Myrmecridium, Fusarium, Talaromyces, Claviceps, Pyrenochaetopsis, Crepidotus, Lycoperdon, Geastrum, Colletotrichum, Kodamaea, Ogataea, Puccinia, Sympodiomycopsis, Orpinomyces, Paraconiothyrium, Pichia, Sporisorium, Cytospora, Purpureocillium, Setophoma, Rhizophagus, Hanseniaspora, Trichosporon, Entrophospora, Dioszegia, Trichoderma, Penicillium, Naganishia, Filobasidium, Aureobasidium, Paraphoma, Strelitziana, Torula, and Vishniacozyma.

Importantly, some aeromicrobes such as Chryseobacterium, Leucobacter, Psychrobacter, Pseudoxanthomonas, Rothia, Candida, Wallemia, Ganoderma, Aspergillus, Fusarium, Trichoderma, Penicillium are present in the aforementioned microorganism. These types of microorganisms have been identified in various environments including litter, soil, plant debris, and even air.

## 4 Discussion

The experimental findings demonstrate a temporal pattern in the pH value and NH_3_-N concentration of the goat rumen fluid following feeding, wherein both initially decrease and subsequently increase. Notably, these two variables exhibit completely aligned trends.

In conjunction with the trend of total volatile fatty acid (TVFA) concentration, it was observed that the TVFA concentration in the goat rumen reached a minimum while the NH_3_-N concentration peaked at 14 h (h0) after morning feeding. The simultaneous increase in NH_3_-N concentration and decrease in TVFA concentration contribute to the resulting high pH value through their interaction. These observations regarding pH value, NH_3_-N concentration, and TVFA concentration are consistent with established biological patterns characteristic of rumen fluid, thereby enhancing the reliability of the experimental data to a certain extent.

The herbivore digestive tract harbors a complex community of anaerobic microbes that collaborate in the degradation of lignocellulose (Henderson et al., 2015; Wallace et al., 2019; Peng et al., 2021). Extensive research has been conducted on rumen microorganisms (Denman et al., 2018; Li et al., 2019; Yu et al., 2022). However, the majority of existing research on rumen microorganisms has focused exclusively on anaerobic microbes due to the anaerobic nature of the rumen habitat and the partial oxidation of substrates (Russell and Rychlik, 2001; Peng et al., 2021). As a result, there is limited literature documenting the presence of aerobic fungi in the rumen (Almeida et al., 2012).

As mentioned in the results section, aerobic fungi such as Aspergillus, Penicillium, and Trichoderma are commonly encountered in litter, soil, plant debris, and air. It is reasonable to speculate that these fungi may enter the rumen through dietary intake. Indeed, our findings have revealed the presence of several species of aerobic fungi, including Aspergillus, Penicillium, Trichoderma, and others, within the goat rumen.

In 1969, Brewer and Taylor made the initial observation of characteristic fungal morphologies belonging to Aspergillus and Spores groups in the rumen fluid of extensively farmed sheep. At that time, the precise role and significance of aerobic fungi in the rumen were not fully recognized (Brewer and Taylor, 1969). James, in his work “Factors That Alter Rumen Microbial Ecology” (2001), also reported the presence of spores and hyphae of obligate aerobic fungi in the rumen (Russell and Rychlik, 2001).

In 2012, Patrícia et al. investigated the microbiota in the rumen fluid of Holstein cows and heifers in Minas Gerais, Brazil. They identified a substantial population of Aspergillus fungi in the rumen. Sampling was carried out before the first meal of the day (Almeida et al., 2012). Furthermore, in 2021, R.F. Neto reported the presence of Rhizopus, within the rumen. This study emphasized the ability of Rhizopus to survive in the unique rumen environment (Fabino Neto et al., 2020).

Figure 2 illustrates the growth of colonies from these microorganisms within an entirely aerobic environment, originating from goat rumen fluid. Although only aerobic fungi were observed in this cultivation, the intriguing experimental results have aroused our suspicion regarding the potential long-term presence of aerobic microorganisms in the goat rumen. These microorganisms may have the ability to survive and proliferate between successive feedings, thus exerting an impact within the goat rumen.

Although the *in vitro* culturing technique employed in our experiment was simplistic, it demonstrated broad applicability and reproducibility. These findings suggest the potential long-term persistence of obligate aerobic microbial communities in the rumen environment, thus prompting further investigations into rumen microbiota diversity. Subsequent experiments employed mature 16 s and ITS testing to identify bacterial and fungal strains in the rumen at different time points after morning feeding. Our results revealed the presence of obligate aerobic bacteria and fungi in the rumen fluid across four distinct time points. Notably, these microorganisms encompassed a range of microbial taxa, including Gordonia, Flavobacterium, Bacillus, Aspergillus, Penicillium, Trichoderma, and other fungi.

In 2020, Elnaz et al. detected aerobic fungi in the rumen contents of cattle through ITS (Azad et al., 2020). The experimental site was in Canada, the experimental animals were Angus steers, the sampling time was immediately after they fed, and the feeding method was grazing. The researchers suggest that these fungi may represent epiphytic fungal populations inhabiting forage and other diets and may not be directly involved in rumen fermentation.

In 2022, our research team detected aerobic fungi within the rumen of buffalo during an experimental study conducted in Yunnan, China (Wang et al., 2022). Rumen fluid samples in this trial were collected 2 h after morning feeding.

Many studies have reported similar findings, including the detection of obligate aerobic fungi within the rumen. However, these results have often been overlooked by researchers for two primary reasons. Firstly, it may have been assumed that these obligate aerobic microorganisms are only transiently present in the rumen and originate from external sources, leading to neglect of their ecological significance within the rumen. Second, techniques for 16 s and ITS detection may be sufficient to detect obligate aerobic microorganisms for a certain period of time after their death (Li et al., 2016; Denman et al., 2018).

For instance, in a study conducted by Xing et al. (2020), Fusarium (12.96%) and Aspergillus (4.54%) fungi were detected within rumen fluid during *in vitro* fermentation experiments. The researchers emphasized the crucial role of these fungi’s enzymatic activities in the biodegradation of lignocellulosic agricultural waste materials (Xing et al., 2020). However, since the main focus of this study was on the microbial degradation of feed components rather than specifically investigating obligate aerobes, the obligate aerobic nature of these fungi was not explicitly examined. In our current study, we have observed the presence of these two obligate aerobic fungi within the rumen, shedding light on their potential roles in rumen ecology. This further prompts inquiries regarding their contributions to feedstuff fermentation.

Over the course of extensive research conducted over many years, the presence of obligate aerobic microorganisms within the rumen has been confirmed by certain researchers through various approaches, such as transcriptome sequencing technology or more direct experiments involving isolation and culture.

In 2020, Zhang et al. utilized transcriptome technology to identify a diverse population of specialized aerobic bacteria thriving within the rumen (Zhang et al., 2020). Samples were collected prior to feeding from three different breeds of animals, namely Angus (AN), Charolais (CH), and Kinsella Composite (KC). The obligate aerobic bacteria identified in this study belonged to Basidiomycota, Mucor, Ascomycota, Chytridiomycota, Mucor, and Unclassified Fungi. Metatranscriptomic datasets were analyzed to identify these fungi, indicating their presence as active microorganisms within the rumen, rather than inactive or dead microbes.

In 2021, Ronaildo Neto et al. isolated and examined 30 different fungi from the sheep rumen microbiome, assessing their production of starch-degrading enzymes across three incubation periods (24, 42, and 72 h). Among the 30 fungal isolates tested, 21 belonged to Aspergillus spp., 6 to Penicillium spp., and 3 to Rhizomucor spp. This study focused on grass-fed sheep in Mato Grosso, Brazil, where it is noteworthy that a majority of the isolated fungi exhibited an aerobic nature (Fabino Neto et al., 2020).

As depicted in Figure 5, we selected four fungi to illustrate the fluctuations in relative abundance within the diverse rumen fungal population. Among these fungi, Aureobasidium is a facultative aerobic organism with a strict dependency on gas for its growth. The figure reveals a substantial decline in the relative abundance of Aureobasidium from 19.80% to approximately 0%, suggesting a gradual decrease in gas levels within the goat rumen following morning feeding. However, the obligatory aerobic fungi, namely Aspergillus, Penicillium, and Fusarium, consistently persisted, suggesting that oxygen may have been present in the goat rumen, but was consumed.

Furthermore, the relative abundance of obligate aerobic fungi changed continuously at 2, 4, 8, and 14 h after morning feeding, with both increases and decreases, indicating that these fungal communities were in a state of survival.

Although there seems to be a contrasting relationship between the gradual decline in gas levels and the persistent presence of oxygen in the rumen, it is noteworthy that the oxygen required for the growth of obligatory aerobic fungi does not necessarily have to exist in a gaseous state. It can also be dissolved within the rumen fluid. Moreover, if the air entering the rumen due to rumination is adequately mixed with the rumen’s digestive contents, it can explain these observations. The physical effects of this mixing process align with the established biology of ruminants.

The apparent contradiction between the gradual decline in rumen gas levels and the persistent presence of oxygen can be elucidated by the fact that gaseous oxygen is not necessarily essential for the growth of obligate aerobic fungi. These fungi can survive in the rumen by being dissolved oxygen in the rumen fluid, as mentioned earlier (Lee et al., 2003).

While previous studies have established a strong correlation between volatile fatty acids (VFAs) and the rumen microbiota (Hong et al., 2004; Hungate1000 project collaborators et al., 2018; Anderson and Fernando, 2021; Glendinning et al., 2021). It is important to note that these studies did not include obligate aerobic microorganisms, which can be considered as “transient microorganisms” in the rumen (Azad et al., 2020; Zhang et al., 2020).

Interestingly, our experiment has demonstrated the existence of obligate aerobic fungi within the goat rumen, providing evidence of a significant association between these microorganisms and VFAs. This correlation has also been observed with obligate aerobic bacteria and fungi, suggesting their potential importance in the goat rumen ecosystem. However, further investigation is required to fully understand the roles of obligate aerobic microorganisms in the rumen.

In 2021, Flavia Oliveira Abrão successfully cultured Rhizopus fungi from the rumen of cows in a study conducted in Minas Gerais, Brazil, employing the captive breeding method. The isolation of these Rhizopus fungal strains has proven to be highly significant, particularly due to the substantial increase in the *in vitro* dry matter digestibility (IVDMD) of cattle upon their introduction. This research demonstrates promising prospects for the development of microbial or probiotic additives to enhance the digestive capacity of cattle feeding on lignified tropical pastures (Abrão et al., 2021).

Another notable study in 2021, led by Janete Maria da Silva Alves, focused on the isolation of mold strains from the rumen of Brazilian beef cattle grazing in Minas Gerais, Brazil. The identification and isolation of obligate aerobic microorganisms from the rumen not only shed light on their potential as industrial microorganisms but also highlighted their prospective use as feed additives for ruminants (da Silva Alves et al., 2021).

Obligate aerobic microorganisms are continuously introduced into the rumen environment from the surrounding ecosystem while the animal is feeding. Throughout this process, the rumen serves as a complex and dynamic culture medium, and the abundance of obligate aerobic microorganisms fluctuates in response to changes in both time and oxygen levels.

Numerous studies have demonstrated that these microorganisms do not have a transient presence but rather persist within the rumen for extended periods, fulfilling significant roles. These roles may include: firstly, the initial breakdown of plant fibers, participation or influence in nutrient decomposition, and the production of volatile fatty acids (see Table 1).

**Table 1:** Nutrient composition of roughage and concentrate feed.

Diet	DM%	CP (% on DM basis)	EE (% on DM basis)	NDF (% on DM basis)	ADF (% on DM basis)	ASH (% on DM basis)	Ca (g/kg on DM basis)	P (g/kg on DM basis)
King grass	29.2	7.74	0.7	44.8	43.3	13.8	1.76	1.47
Corn flour	78.2	12.4	2.4	28.7	25.7	7.2	2.02	1.17

Secondly, the consumption of oxygen during early fermentation stages creates anaerobic conditions for anaerobic microorganisms to ferment. In the rumen ecosystem, oxygen consumption is an important factor that inhibits the continuous growth and proliferation of microorganisms such as mold, presenting high significance. Simultaneously, some bacteria display pesticide-degrading capabilities. In 2022, Zhao et al. isolated a β-cypermethrin-degrading bacterium, named GW-01, from sheep rumen food boluses which showed significant degradation of pyrethroid pesticides (Zhao et al., 2022).

Aerobic fungi also play a vital role in maintaining a relatively anaerobic environment within the rumen. Despite the decrease in the abundance of obligate aerobic microorganisms as the oxygen concentration decreases, they persist and continue to consume oxygen during the processes of feeding and rumination within the rumen.

## 5 Conclusion

In conclusion, our findings suggest that obligate aerobic microorganisms survive in the goat rumen over time, with their relative abundance varying with diet feeding cycles. The aerobic microorganisms found in the rumen of goats were Aspergillus, Penicillium, and Fusarium, Flavobacterium, Nocardiopsis, and Moraxella. We have also identified correlations between Fretibacterium, Aspergillus, Fusarium, and volatile fatty acids.

Our proposed hypothesis that aerobic microorganisms are constantly present in the rumen can be further explored in future studies. These findings provide insights into the dynamic microbial ecosystem of the goat rumen. Further research on rumination behavior and its effect on the relative abundance of aerobic microorganisms in the rumen will help to enhance our understanding of this complex ecological system.

## Data availability statement

The original contributions presented in the study are publicly available. This data can be found at: http://www.ncbi.nlm.nih.gov/bioproject/996308. BioProject ID: PRJNA996308. http://www.ncbi.nlm.nih.gov/bioproject/997758. BioProject ID: PRJNA997758.

## Ethics statement

The animal study was approved by The U.K. Animals (Scientific Procedures) Act, 1986. The study was conducted in accordance with the local legislation and institutional requirements.

## Author contributions

RW: Conceptualization, Data curation, Writing – original draft. DH: Conceptualization, Validation, Writing – review & editing. CC: Methodology, Validation, Writing – review & editing. DS: Formal analysis, Validation, Writing – review & editing. HP: Formal analysis, Supervision, Writing – review & editing. MH: Project administration, Supervision, Writing – review & editing. XH: Data curation, Formal analysis, Writing – review & editing. ZH: Data curation, Methodology, Writing – review & editing. BW: Methodology, Validation, Writing – review & editing. HL: Formal analysis, Investigation, Writing – review & editing. PT: Funding acquisition, Supervision, Writing – review & editing.
